# Measuring the Efficacy of Myofascial Rollers and Post-isometric Relaxation Technique in Relieving Pain Intensity and Postural Deviation Using Plumb Line Assessment for the Treatment of Upper Cross Syndrome in Dental Undergraduate (UG) Students

**DOI:** 10.7759/cureus.29831

**Published:** 2022-10-02

**Authors:** Anam R Sasun, Swapna Jawade, Neha Chitale

**Affiliations:** 1 Department of Physiotherapy, Ravi Nair Physiotherapy College, Datta Meghe Institute of Medical Sciences, Wardha, IND; 2 Department of Musculoskeletal Physiotherapy, Ravi Nair Physiotherapy College, Datta Meghe Institute of Medical Sciences, Wardha, IND

**Keywords:** plumb line assessment, original article, physiotherapy rehabilitation, pain, post-isometric relaxation, myofascial rollers, dental undergraduates, upper cross syndrome

## Abstract

Background: In today’s world, the upper cross syndrome is growing more common and becoming very prevalent among dental undergraduate practitioners. One of the most important conditions for which dentists seek physiotherapy treatment is neck pain. It is characterized by overactive pectoralis and trapezius muscles. It is frequently linked to poor posture in dental students' daily life, causing them to miss their work.

Objectives: The first objective of our study was to find the efficacy of myofascial rollers and post-isometric relaxation technique along with conventional therapy for pain relief and correction of postural deviation in undergraduate dental students. And, the second objective of the study was to compare the effect of myofascial rollers and post-isometric relaxation techniques in upper cross syndrome.

Methods: The study was conducted with pre-test and post-test methods. The study consisted of 80 participants who were included based on our inclusion and exclusion criteria. The study sample was randomly assigned into two groups. Each group consisted of a total of 40 participants. Group A was treated using myofascial rollers and hot packs, and Group B was treated using the post-isometric relaxation technique and hot packs. Patients were asked to mark their intensity of pain on the Numerical Pain Rating Scale and an assessment of postural deviations (in mm) was noted through a plumb line in the posture grid. Posture assessment was done in lateral view. The protocol covered four weeks of treatment based on the defined protocol. Finally, the t-square test and Chi-square test were used to compare the difference in the result. Also, the level of significance was kept at <0.05.

Result: Statistical analysis was done using descriptive and inferential statistics using student paired, unpaired, and chi-square test. IBM SPSS Statistics for Windows, Version 27.0 (Released 2020; IBM Corp., Armonk, New York, United States) was used. The Numerical Pain Rating Scale showed mean deviations of (4.15±1.29) for Group A and (3.30±1.01) for Group B. Plumb line assessment showed mean deviations of (9.09±4.31) for Group A and (6.33±2.36) for Group B. Also, Numerical Pain Rating Scale showed (t=3.26, p=0.002) and Plumb line deviation showed (t=3.57, p=0.001). Conclusion: Through our study, we conclude that statistically no significant differences were found in pre-intervention and post-intervention, but myofascial rollers gave better results as compared to the post-isometric relaxation technique in alleviating pain and correcting postural deviation.

## Introduction

Musculoskeletal disorders are the leading cause of disability among people aged 20 to 50 years and are primarily related to their jobs, with headaches and chronic neck pain being the most common complaints worldwide as per WHO literature [1]. An upper cross syndrome is caused by weak lower and middle trapezius, tight upper trapezius and levator scapulae; weak deep-neck flexors, tight sub-occipital muscles, and sternocleidomastoid, weak serratus anterior, and tight pectoralis major and minor [2]. The upper cross syndrome is caused by postures that favour the flexors [3]. The activities and the work of dentists are all related to standing continuously or improperly sitting for long hours [4]. All awkward chair positions cause continuous bending of the neck downwards as well as continuous bending of the spinal cord, which causes continuous stress on anatomical structures. As a result, dentists are more prone to developing problems and upper cross syndrome. According to a text review of musculoskeletal problems among dental professionals, the incidence rate of general musculoskeletal pain among dentists stretches between 64% to 93%, with the most widely stated location of pain being the back with 36.3% to 60.1% and the neck being 19.8% to 85% [5]. 

These stances are associated with differences in the position of the scapula. Overactive muscles and varied kinematics around the neck and shoulder muscles cause gradual changes in muscular tension. Thoracic-four (T4) syndrome has been coupled with back pain along with difficulty in apt functioning and generalized weakness, and other upper body symptoms. Also, T4 syndrome is known to induce chest pain and pseudo angina [6]. Physical therapists have worked in various industrial fields for work movement analysis and educational programs to prevent work-related musculoskeletal disorders as a result of this legislation. As a result, the areas in which physical therapists work have also been expanded. Hand and arm disorders, such as carpal tunnel syndrome and tendonitis, are the most common reasons for dentists and dental hygienists to retire due to disability [7]. Patients suffering from weakness of neck muscles have reported having motor control deficits. As cervical spine is a more sensitive area of the spinal column than the thoracic spine because of its role in safeguarding important nerves and blood vessels [8]. Performing rapid and high-velocity vertebral rotations induce a risk of injury to vertebral arteries, muscle spasms, and Wallenberg syndrome. Because of numerous risks associated with such an approach. A thoracic spine approach should be practised and considered [9].

Physiotherapy practitioners have been involved in a number of industrial settings to understand work movement patterns and establish appropriate policies to help minimize work-related musculoskeletal disorders as a result of this legislation. Following a thorough review of available texts, several works of literature were found that stated post-isometric relaxation technique as an effective method to treat neck pain and myofascial rollers were found less effective on pain intensity [10]. Therefore, this study was conducted to study the effect of myofascial rollers and post-isometric relaxation techniques for reducing pain intensity and posture deviations in upper cross syndrome in dental undergraduates.

## Materials and methods

**Figure 1:**
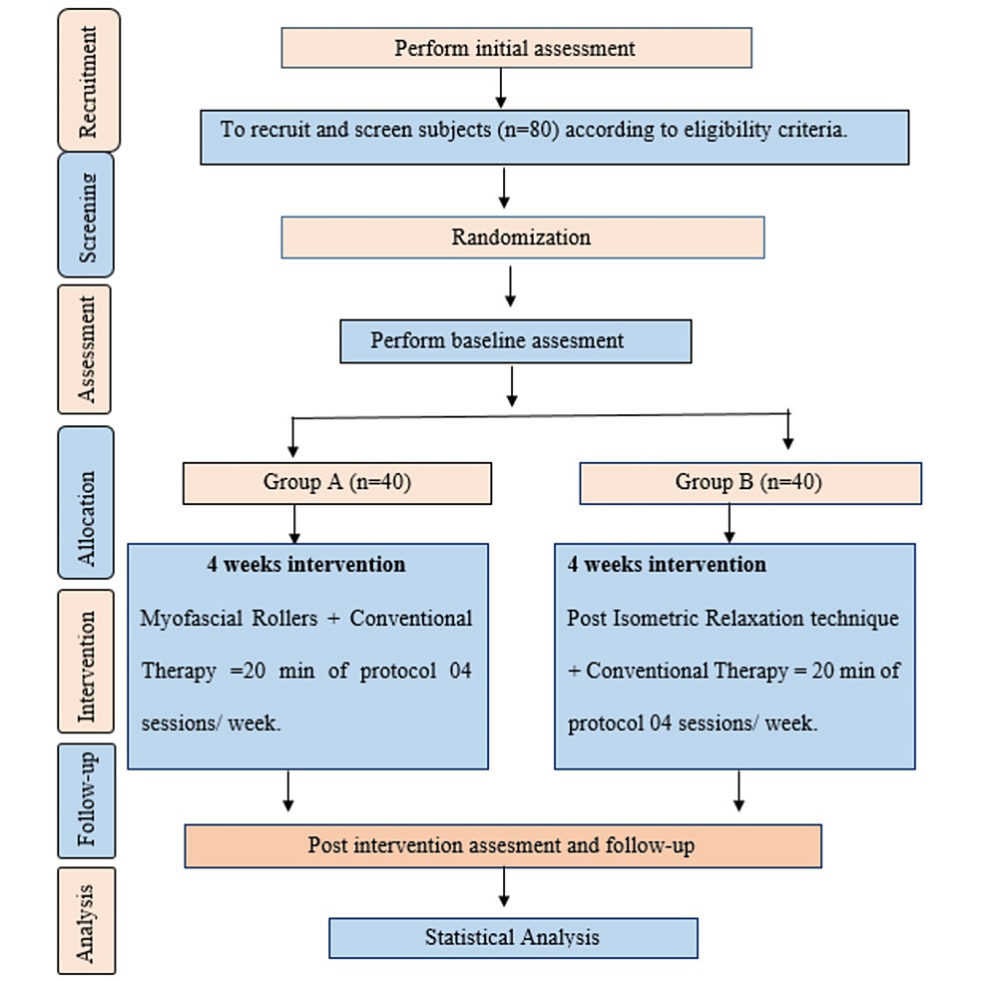
Flow chart of the study n=number of participants

After receiving approval from the Institutional Ethics Committee of Datta Meghe Institute of Medical Sciences, Deemed to be University, with Ethical Clearance Number DMIMS(DU)/IEC/2021/381), the study was conducted in the musculoskeletal physiotherapy out-patient department of Ravi Nair Physiotherapy College, Sawangi (Meghe), Wardha, India. The inclusion criteria of our study included both male and female dental students of 18-25 years of age, participants working at clinics for at least two years, subjects constantly or frequently suffering from neck-shoulder pain, and those willing to participate in the study. The exclusion criteria included age more than 25 years, those diagnosed with malignancies of soft tissues and joints, congenital neck and shoulder deformities, recent fractures, unstable cardiovascular pathologies, and patients on another clinical trial. The participants were explained the objectives and approaches of the study, and written patient consent forms were signed by them. The participants with musculoskeletal pain due to upper cross syndrome were randomized through simple random sampling and were allocated as randomly those who fulfilled the inclusion criteria into group A and group B. Randomization and allocation were done by the primary researcher, who is an undergraduate student of physiotherapy. Outcome measures were assessed before the beginning of the study, and after the completion of the study, by the same undergraduate student of physiotherapy. Randomization was done based on inclusion and exclusion criteria along with a simple random sampling method and allocated through SNOSE (sequentially numbered, opaque sealed envelope) method. The envelopes were kept with serial numbers. The collected data was stored in a safe, locked area with restricted access, for a biostatistician for further analysis. The registration, treatment, and evaluation schedules for the study bonded to the guidelines in the standard protocol items: a suggestion for conducting intervention trials [11]. The flow chart of the study is described in Figure 1.

Outcome measures

A physiotherapy undergraduate student who was aware of the study but blinded to the intervention evaluated the following outcome measures before and after the intervention.

Numeric Pain Rating Scale (NPRS)

It is a uni-dimensional scale to measure pain intensity, particularly in adults. It is a 10-rating scale and an 11-point numeric scale in the form of a horizontal bar or line. Zero is no pain and 10 is intolerable/ worst pain. A therapist can ask questions verbally or it can be self-scored. It has several merits, like it takes less than one minute to complete, and can be understood in any language; i.e. there is no language barrier. It is a valid and reliable scale that measures pain intensity and experience [12]. *Plumb Line Assessment*

While assessing posture symmetry, rotations should be observed in three views: anterior, posterior, and, lateral. Look for alignment of the head, curvatures of the spine, and shoulder symmetry followed by pelvic, hip, and ankle. This research assessment of posture will be done in a lateral view [13].

Intervention

**Figure 2:**
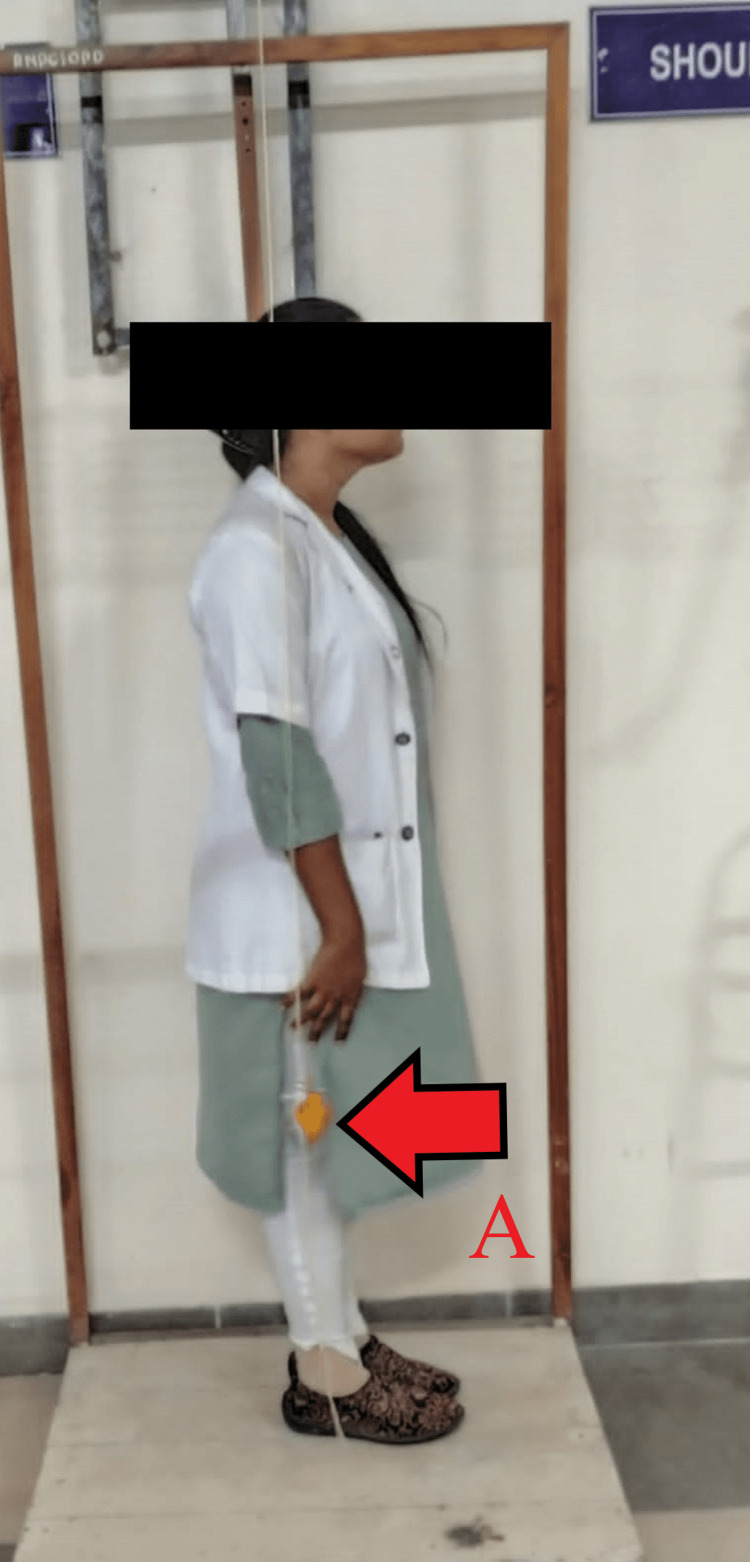
Plumb line assesment on lateral view of patient (red arrow denoting plumb bob of posture grid) A: plumb bob on posture grid

Two groups were assembled. Demographic data were collected from the patient and each participant underwent the measurement of NPRS score and plumb line assessment (Figure 2). During the time period of training, all participants were told not to alter their current physical activities. The participants in both groups received treatment for four weeks each.

**Figure 3:**
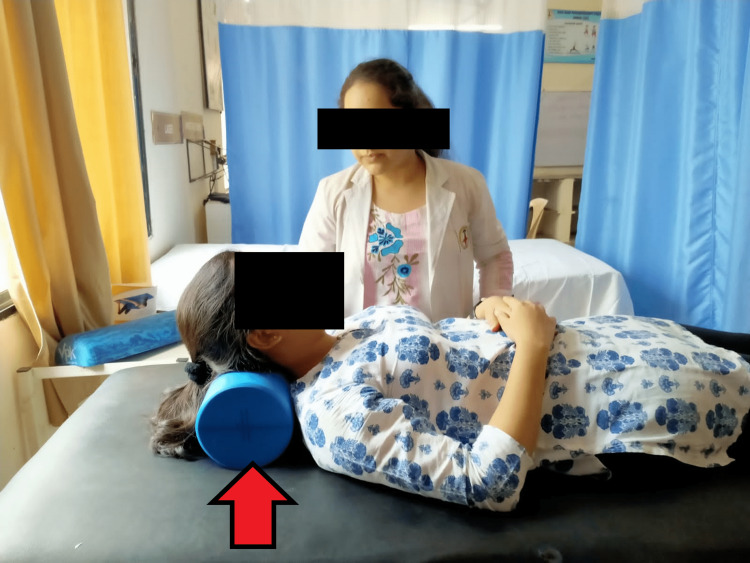
Application of myofascial rollers to patient (red arrow denoting myofascial roller being used)

Group A (Myofascial Rollers and Conventional Therapy):

The participants in this group underwent treatment through myofascial rollers and conventional therapy. Prior to treatment thorough physiotherapy assessment was done for palpating tenderness, trigger points, and taut bands followed by conventional therapy including the application of hot packs for a period of 15 minutes and subsequently, application of myofascial rollers. Myofascial rollers were rolled on the trapezius, levator scapulae, rhomboid major, and rhomboid minor muscles. The rollers were moved in all directions along the length of a particular muscle. The application time of the roller was 10-15 seconds followed by a relaxation time of 10-12 seconds. For better results and comfort of the patient, treatment was given in a supine lying position. Each session lasted for 20 minutes for four days per week for a duration of four weeks (Figure 3).

Group- B (Post-isometric Relaxation and Conventional Therapy)

**Figure 4:**
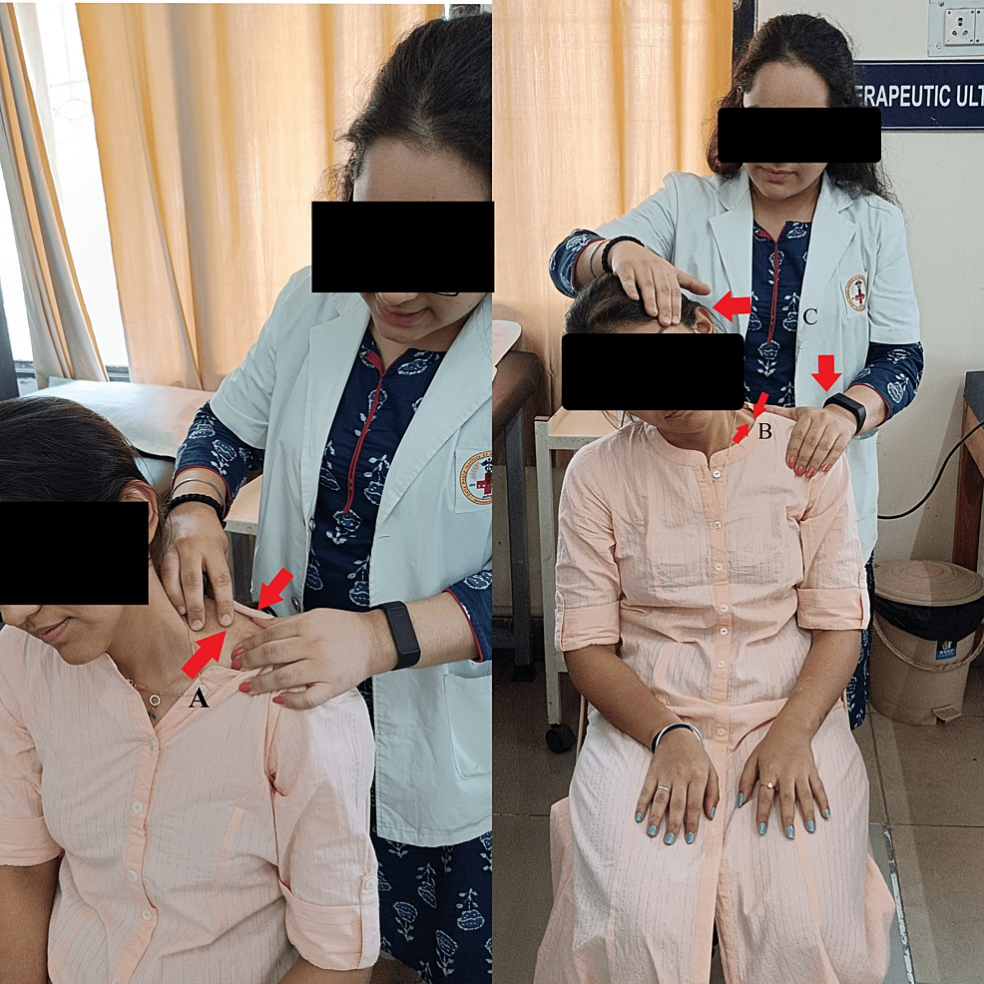
A: palpating for tenderness and trigger points (two red arrows denoting the area of palpation), B: showing stretching to trapezius muscle (two red arrows denoting lengthened muscle), and C: showing post-isometric relaxation technique

The participants in this group underwent treatment with the post-isometric relaxation technique. Prior to treatment, a thorough physiotherapy assessment was done for palpating tenderness and taut bands followed by conventional therapy including the application of hot packs for a period of 15 minutes. Post-isometric relaxation was given for five repetitions, for the trapezius, levator scapulae, and rhomboid, both major and minor muscles. Gentle stretching of the trapezius muscle was given to the patient. The subject was in a sitting position with their back supported and their head tilted towards one side. The patient was asked to contract their muscles for 8-12 seconds along with a gentle stretch to the neck, followed by a relaxation duration of 10-12 seconds. Likewise, five barriers were achieved. The patient was instructed not to hold breaths (Figure 4).

## Results

**Figure 5:**
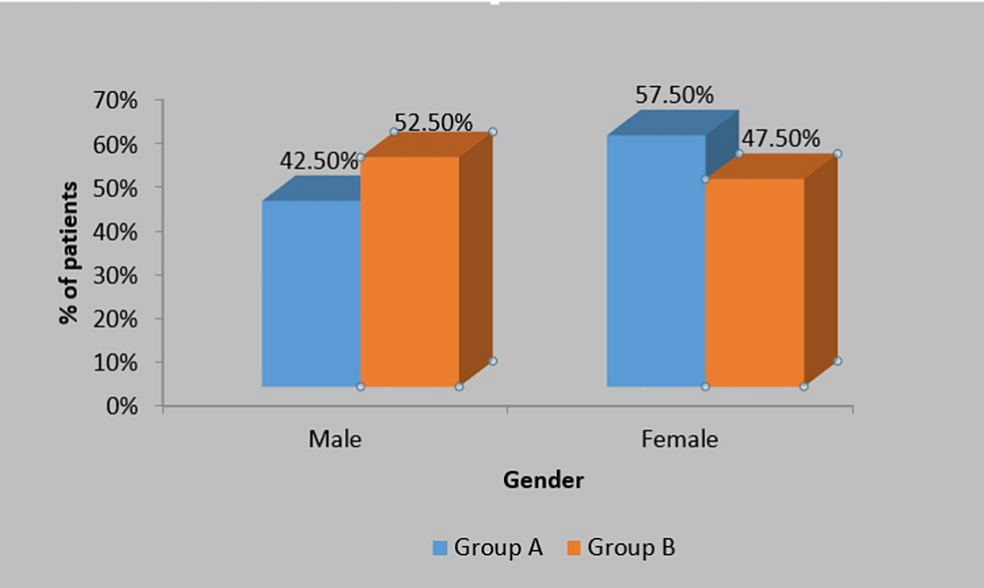
Distribution of patients according to gender

The level of significance for the statistical analysis was set at p<0.05, and descriptive and inferential statistics were performed using the chi-square test, student paired and unpaired t-tests, and software from IBM SPSS Statistics for Windows, Version 27.0 (Released 2020; IBM Corp., Armonk, New York, United States) and GraphPad Prism 7.0 (GraphPad Software, San Diego, California). The mean age of the patients of group A was 22.95±1.66 and in group B it was 22.85±1.81 and the difference between the two groups is statistically not significant. In group A, 42.5% of the patients and in group B, 52.5% were males, and 57.5% of the patients in group A and 47.5% in group B were females; the difference between the two groups was statistically not significant (Table 1; Figure 5).

**Table 1:** Distribution of patients according to baseline characteristics NS: Non -Significant

Baseline characteristics	Group A	Group B	p-value
Age in years	22.95±1.66	22.85±1.81	0.79, NS
Gender			
Male	17(42.5%)	21(52.5%)	0.65, NS
Female	23(57.5%)	19(47.5%)	

**Figure 6:**
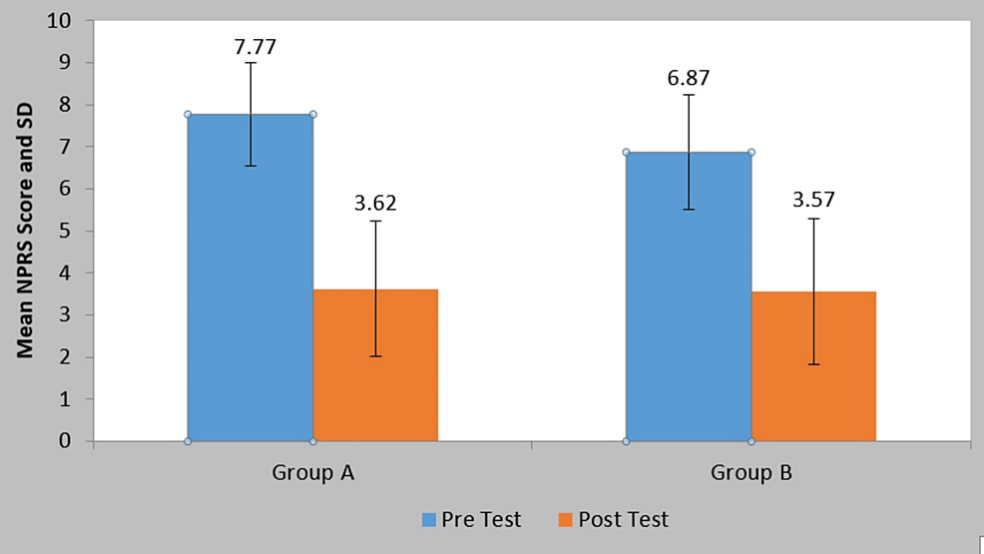
Comparison of NPRS score in two groups at pre- and post-treatment SD=standard deviation; NPRS=Numerical Pain Rating Scale

The mean NPRS score in group A at pre-treatment was 7.77±1.22 and at post-treatment, it was 3.62±1.61. By using the Student’s paired t-test, a statistically significant difference was found in NPRS scores at pre and post-treatment (t=20.31, p=0.0001). The mean NPRS score in group B at pre-treatment was 6.87±1.36 and at post-treatment, it was 3.57±1.73. By using the Student’s paired t-test, a statistically significant difference was found in NPRS scores at pre- and post-treatment (t=20.50, p=0.0001). On comparing the mean difference in NPRS scores among patients of two groups statistically, a significant difference was found in NPRS scores among patients of two groups (t=3.26, p-value=0.002) (Table 2; Figure 6).

**Table 2:** Comparison of NPRS score in two groups at pre- and post-treatment S=Significant; NPRS=Numerical Pain Rating Scale

Group	Pre Test	Post Test	Mean Difference	Student’s Paired t-test value
Group A	7.77±1.22	3.62±1.61	4.15±1.29	20.31 P=0.0001, S
Group B	6.87±1.36	3.57±1.73	3.30±1.01	20.50 P=0.0001, S
Comparison of mean difference in two groups (Student’s unpaired t-test)→			t-value	p-value
			3.26	0.002, S

**Figure 7:**
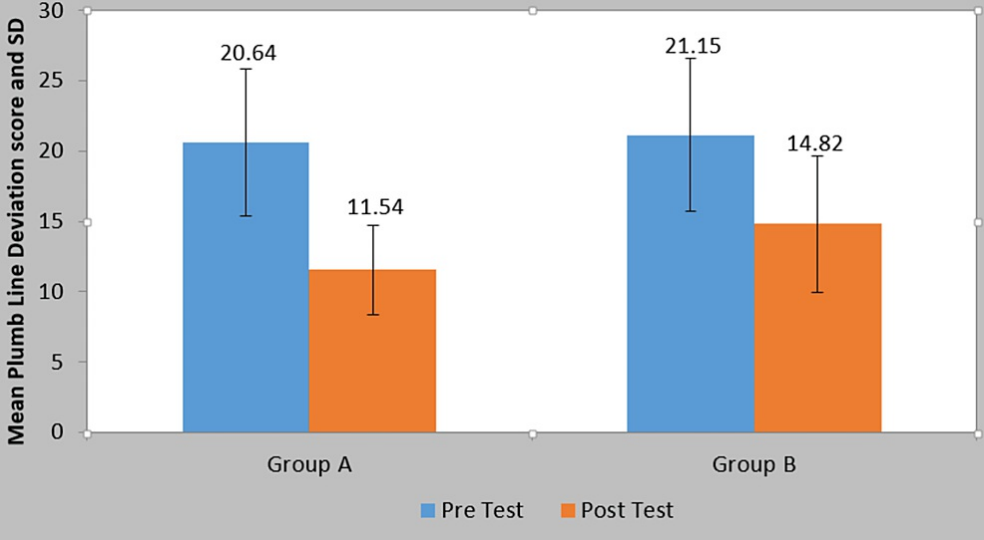
Comparison of plumb line deviation score in two groups at pre- and post-treatment SD=Standard deviation

The mean plumb line deviation score in group A at pre-treatment was 20.64±5.24 and at post-treatment, it was 11.54±3.15. By using the Student’s paired t-test statistically significant difference was found in the plumb line deviation score at pre- and post-treatment (t=13.33, p=0.0001). The mean plumb line deviation score in group B at pre-treatment was 21.15±5.43 and at post-treatment, it was 14.82±4.84. By using the Student’s paired t-test, a statistically significant difference was found in the plumb line deviation score at pre- and post-treatment (t=16.91, p=0.0001). On comparing the mean difference in the plumb line deviation score among patients of two groups statistically, a significant difference was found in the plumb line deviation score among patients of two groups (t=3.57, p-value=0.001) (Table 3; Figure 7).

**Table 3:** Comparison of plumb line deviation score in two groups at pre- and post-treatment S=significant

Group	Pre Test	Post Test	Mean Difference	Student’s Paired t-test value
Group A	20.64±5.24	11.54±3.15	9.09±4.31	13.33 P=0.0001, S
Group B	21.15±5.43	14.82±4.84	6.33±2.36	16.91 P=0.0001, S
Comparison of mean difference in two groups(Student’s unpaired t-test)→			t-value	p-value

## Discussion

This study was conducted to find the efficacy of myofascial rollers and post-isometric relaxation techniques using plumb line assessment for the treatment of upper cross syndrome in undergraduate (UG) dental students. Myofascial release (progressive pressure release; PPR) is a technique for improving the range of motion (ROM) and reducing muscle tension by enlarging the sarcomere within muscle fibres by gradually applying pressure to the motor trigger point (MTrP) before the specialist feels the tension release [14]. Physiotherapeutic recommendations are being made for dentists to avoid the onset of musculoskeletal pain, which include performing enough neck extension exercises, straightening the lower back from awkward positions, and tilting the head back until they can see the ceiling, then moving their head six to ten times to cause their jaw to touch the acromion. Exercises to strengthen the muscles at the back of the neck are also important. Shoulder and upper-extremity exercises help with neck pain. As a result, posture-related damage can be avoided by doing exercises like turning the shoulder after each treatment [15]. Both eccentric muscle techniques and stretching exercises were equally effective in reducing neck pain and increasing the ROM of the neck [16]. Myofascial was found to enhance vertical alignment of muscles along with the lengthening of the body which provides more space for the proper functioning of osseous structures, nerves, muscles, blood vessels, and organs which improves the function [17]. It is a gentle load exerted on fascial restrictions that engenders the vasomotor response and tends to increase the circulation area, thereby enhancing lymphatic drainage of toxic metabolic wastes. It realigns the fascial planes and, more importantly, resets the soft tissue proprioceptive sensory mechanisms. This component later recalibrates the central nervous system, enabling a normal functional ROM without eliciting the old pain trend [18].

Chu and Butler in 2021 presented a case that described the consequences of poor postural positions for a prolonged period on gastroesophageal reflux disease (GERD) [19]. The physiological effect of the upper cross syndrome is that patients can experience heartburn due to acid reflux caused by prolonged and extended sitting periods. Performing breathing exercises can help patients with forward head posture. When comparing changes within groups, it was revealed that sternocleidomastoid and scalene activities increased in both groups. When further changes were compared, sternocleidomastoid muscle performed exceptionally well. Forward head posture causes severely exaggerated tension and contraction of neck muscles, leading to decreased relaxation and hampered contraction. Muscle activities increase as a result of stiffness in the neck flexors [20]. Patterns to uphold correct posture are regarded as necessary in relation to cervical spine postures. Thus, avoiding damage and pain [21]. Pain reduction was substantially responsible for changes in trigger point status from active to latent or resolved period. Pain relief was significantly linked to improvements in cervical spine bending and rotation, and patients reported improved physical and emotional well-being, mood, and impairment decline [22]. National Academy of Sports Medicine (NASM) exercises combined with ergonomics treatments could be an excellent rehabilitation session [23]. The Comprehensive Corrective Exercise program for people with the upper cross syndrome is viable, and it leads to benefits in muscle imbalances and postural alignment that last even after short-term detraining and can be used as a tactic for correcting postural malalignments. Muscular re-education and manual therapy to the spine proved more effective in reducing the intensity of pain [24].

Thoracic manipulation is more beneficial for reducing neck pain. And mechanical manipulations benefitted patients in reducing neck disability. Hand and arm disorders, such as carpal tunnel syndrome and tendonitis, are the most common reasons for dentists and dental hygienists to retire due to disability. Practising high velocity oriented approaches like vertebral rotations induces a high probability of risks for internal structures of the area, like injury to the vertebral artery. Therefore, it is advised to practice such approaches with the thoracic spine [25]. A combination of re-educating the muscle and manual therapy is more effective than using these techniques singularly. Also, there are shreds of evidence in the article which suggest chiropractic manipulations along with muscle re-education as an effective method to treat upper cross syndrome [26]. There is evidence in the literature that emphasizes the importance of the eight-week stretching protocol for deep neck flexors. Stretching along with neck exercises is known to enhance strength and endurance. It is also known to improve posture in students. Reduction in temperature of posterior neck muscles may help to improve upper cross syndrome [27]. To ensure the progress movement pattern and control load progression, a laptop monitored by a qualified corrective exercises expert was used. The workplace group received diaries with detailed written and pictorial descriptions which emphasized constantly evaluating the progress, safety, and performances of participants. Individuals were given an exercise program to help with their spontaneous postural changes, muscle activation, movement patterns, and lack of scapula stabilization [28]. An instant improvement is required to correct posture in working dentists, particularly those involved in repairing and extracting second molar maxillary teeth. Due to this improper posturing, such dentists are more prone to develop neck and back pains which reportedly get worsened due to negligence [29].

The reason behind the selection of our research topic was the increasing number of UG dental professionals reporting at our physiotherapy outpatient departments (OPDs) with complaints of neck and shoulder pain with high intensities. This report is unique in that several articles were found that stated post-isometric technique as an effective protocol to treat pain associated with the upper cross syndrome, but no previous article was found that has done a study to find the efficacy of myofascial rollers and post-isometric relaxation techniques using plumb line assessment for the upper cross syndrome. Myofascial rollers gave better results in NPRS score as compared to post-isometric relaxation technique in alleviating musculoskeletal pain associated with neck and shoulder. The limitations of the study were a smaller sample of only 80. For generalizing results, larger-scale studies are required. The small duration of the study was another limitation. Also, there were clothing issues in females during the plumb line assessment. Further studies should be conducted with long intervention timings. This study was conducted only on dental undergraduates; similar studies should be conducted in other occupations.

## Conclusions

In today's era, increasing sedentary lifestyles and lack of exercise are making people suffer from musculoskeletal pain. The major reason behind conducting this study was dental professionals reporting to our physiotherapy hub complaining of musculoskeletal neck and shoulder pain. All awkward chair positions and continuous stress on anatomical structures are making them very prone to developing problems and upper cross syndrome. The findings of our study confirmed that myofascial rollers were more effective than post-isometric relaxation techniques in reducing pain intensity and correcting postural deviations in dental undergraduates suffering from the upper cross syndrome. After the study, participants were educated about the maintenance of healthy postures and repeated stretching intervals in short breaks from work. Both the participating groups showed improvement in pain when results were documented and analyzed after the completion of the intervention duration of four weeks. For generalizing results, larger-scale studies are required. Also, similar studies should be conducted with long intervention timings. This study was conducted only on dental undergraduates; similar studies should be conducted in other occupations to know about their forthcoming risk factors.
